# Catch Muscle Myorod Modulates ATPase Activity of Myosin in a Phosphorylation-Dependent Way

**DOI:** 10.1371/journal.pone.0125379

**Published:** 2015-04-27

**Authors:** Oleg S. Matusovsky, Ulyana V. Shevchenko, Galina G. Matusovskaya, Apolinary Sobieszek, Anna V. Dobrzhanskaya, Nikolay S. Shelud’ko

**Affiliations:** 1 A.V. Zhirmunsky Institute of Marine Biology, Far East Branch of the Russian Academy of Sciences, Vladivostok, Russia; 2 School of Biomedicine, Far Eastern Federal University, Vladivostok, Russia; 3 Institute for Biomedical Aging Research, Austrian Academy of Sciences, Innsbruck, Austria; University of Minnesota, UNITED STATES

## Abstract

Myorod is expressed exclusively in molluscan catch muscle and localizes on the surface of thick filaments together with twitchin and myosin. Myorod is an alternatively spliced product of the myosin heavy-chain gene that contains the C-terminal rod part of myosin and a unique N-terminal domain. The unique domain is a target for phosphorylation by gizzard smooth myosin light chain kinase (smMLCK) and, perhaps, molluscan twitchin, which contains a MLCK-like domain. To elucidate the role of myorod and its phosphorylation in the catch muscle, the effect of chromatographically purified myorod on the actin-activated Mg^2+^-ATPase activity of myosin was studied. We found that phosphorylation at the N-terminus of myorod potentiated the actin-activated Mg^2+^-ATPase activity of mussel and rabbit myosins. This potentiation occurred only if myorod was phosphorylated and introduced into the ATPase assay as a co-filament with myosin. We suggest that myorod could be related to the catch state, a function specific to molluscan muscle.

## Introduction

The smooth muscle of molluscs exhibit the so-called “catch state”, where high tension is maintained for a long time with little expenditure of energy. Molluscan smooth muscle contains a unique composition of thick filament-associated proteins. The major component of large diameter molluscan thick filaments is paramyosin whose surface is covered with myosin [1], twitchin [2, 3] and myorod [4]. Myorod is a protein that is specific to catch muscle [4, 5], its content greatly exceeds the amount of twitchin while it is equally as abundant as myosin [6, 7].

Myorod, a water-insoluble, heat-resistant protein consists of two polypeptides of 112 and 120 kDa [4, 7], is an alternative product of the myosin heavy-chain gene. It contains a C-terminal rod part, which is identical to the rod portion of myosin, and a unique N-terminal domain [8]. The identity of the C-terminal parts and the similarity between the domain structures of myosin and myorod [9] suggest that myorod is integrated into the surface of the paramyosin core in the same manner as myosin, i.e. the myorod “tail” domain (identical to light meromyosin of myosin) predominantly localizes to the surface of thick filaments, while the myorod N-terminus protrudes into the interfilament space. Such a configuration would indicate that the unknown function of myorod might be to influence the interaction between thick and thin filaments. This suggestion is complemented by the analysis of myorod distribution across different muscles in bivalves: so far myorod was found only in muscles capable of catch contraction [4, 5].

Myorod, as well as myosin, is capable of polymerization in solutions of low and physiological ionic strength; however, the properties and structure of myorod filaments differ significantly from those of myosin [7]. In particular, solutions of myorod have unusually high viscosity, far lower aggregation capacity and resistance to MgATP, which differently than for myosin does not suppress polymerization of myorod [7]. Such differences are surprising because both proteins have identical C-terminal rod parts [8] and should therefore possess identical assembly competence domains (ACD), which are responsible for their polymerization [10]. As it turns out, the N-terminal domain of myorod may affect the properties of the C-terminal parts of the molecule and, in turn, its filament assembly [7, 11].

Using *in vitro* assays, it was found that myorod was able to interact with paramyosin, myosin, F-actin and twitchin [4, 7, 12, 13], however, the functional consequences of these interactions are not clear. One intriguing approach to investigate the function of myorod involves twitchin, a giant regulatory protein of molluscan smooth muscle [2]. This protein, as other members of the immunoglobulin superfamily, has a kinase domain near the C-terminus that is highly homologous to the catalytic domain of smMLCK [14]. Immunogold labeling showed that the kinase domain of molluscan twitchin localizes in a regular manner along thick filaments [15]. This would be in accordance with the possibility of a direct interaction of the myorod N-terminus with twitchin’s MLCK-homologous domain *in vivo*.

The unique N-terminal domain of myorod contains several putative phosphorylation sites. One site has been established for smMLCK, which phosphorylates myorod in Thr141 position [16]. A first goal of this study was to clarify the role of N-terminal phosphorylation. To this end, we investigated the effect of N-terminal phosphorylation of myorod on the mechano-chemical properties of the natural protein complex consisting of myosin, myorod and twitchin. It has also been proposed that N-terminus phosphorylation of myorod within this complex could modulate the actin-activated Mg^2+^-ATPase activity of myosin [17]. We showed that indeed N-terminal phosphorylation of myorod potentiates Mg^2+^-ATPase activity of myosin. We further demonstrated that the formation of co-filaments assembled from myosin and phosphorylated myorod is a precondition for the potentiation of myosin Mg^2+^-ATPase activity.

## Materials and Methods

All animal studies were conducted in accordance with institutional guidelines.

### Protein preparation

**Fig 1:**
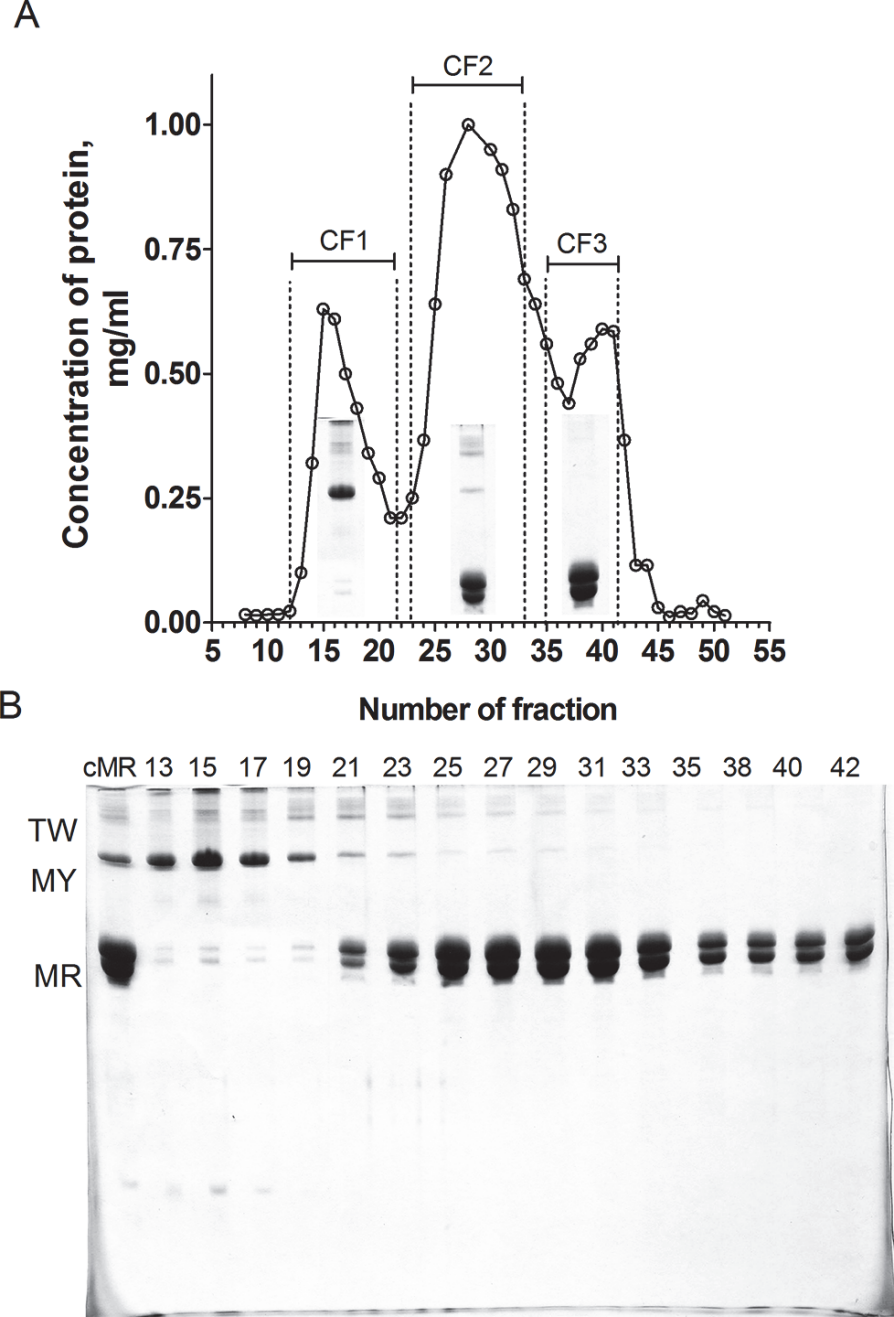
Purification of myorod. (A) Elution profile from Sepharose CL 6B gel-filtration chromatography (B) Samples from column after being subjected to SDS-PAGE (7.7% acrylamide). Selected fractions from each peak were combined and dialyzed. cMR (crude myorod), applied sample; TW, twitchin; MY, myosin; MR, myorod.

Myorod and myosin were prepared from the posterior adductor of *Crenomytilus grayanus* (mussel) as described in our previous works [7, 18]. In the case of myorod, the final purification stage was modified. The 33% to 43% ammonium sulfate fraction [18] was dissolved in solution containing (in mM): 500 KCl, 2 EDTA, 2 NaN_3_, 0.5 PMSF, 0.05 leupeptin, 1 DTT, 50 Tris-HCl, pH 8.0. The fraction was dialyzed against the following solution (in mM): 75 KCl, 2 EDTA, 2 NaN_3_, 5 β-Mercaptoethanol, 50 Tris-HCl, pH 8.0 and precipitated at 15000×*g* for 30 min. The pellet was dissolved in urea buffer containing 6 M urea and (in mM): 600 KCl, 1 NaN_3_, 2 DTT, 0.5 EGTA, 50 Tris-HCl, pH 8.0, and clarified at 25,000×*g* for 30 min. The supernatant (50 mg of total protein) was loaded onto a gel-filtration chromatography column (Sepharose CL-6B, Sigma, 60 cm) equilibrated by the same urea buffer. Fractions from the first peak (CF1, tubes 12–21), from the second peak (CF2, tubes 23–33) and from the third peak (CF3, tubes 35–41) (**Fig 1A**) were selected by SDS–PAGE (**Fig 1B**), combined and exhaustively dialyzed at 4°C against a solution containing (in mM): 30 KCl, 2 NaN_3_, 2 DTT, 1 MgCl_2_, 20 imidazole-HCl, pH 6.5.

Rabbit skeletal tissues were donated from the vivarium of the G.B. Elyakov Pacific Institute of Bioorganic Chemistry (Vladivostok, Russia). All procedures were approved by the Animal Care Committee of A.V. Zhirmunsky Institute of Marine Biology, Far East Branch of the Russian Academy of Sciences (Protocol N 21 from 08.09.2014). Rabbit skeletal actin was prepared as described previously [12] and was freeze-dried with 10% sucrose as F-actin. Rabbit skeletal muscle myosin was prepared according to Margossian and Lowey [19].

Protein concentrations were determined either by microbiuret method or by Bradford method calibrated with *Crenomytilus grayanus* myosin and myorod as described [7].

### Preparation of actomyosin in presence or absence of myorod

The actomyosin reconstituted from rabbit F-actin and rabbit or mussel myosin in presence or absence of unphosphorylated (UnPhos) or phosphorylated (Phos) myorod was prepared in two modes. In the first case, filaments of myosin and myorod were preformed separately under the same conditions by quick dilution of proteins from solution A (in mM): 500 KCl, 2 MgCl_2_, 0.2 EGTA, 1 DTT to 100 mM KCl by solution B containing (in mM): 2 MgCl_2_, 2 NaN_3_, 0.3 CaCl_2_, 20 imidazole-HCl, pH 7.0. The preformed myosin filaments were then mixed with F-actin and preformed UnPhos or Phos myorod filaments and incubated for 20 min at 4°C.

In the second mode AM was prepared by mixing of myosin and F-actin in high ionic strength (solution A) in the presence or absence of UnPhosMR or PhosMR. The mixtures then were diluted to 100 mM KCl by solution B and were incubated for 20 min at 4°C. It was shown that in this particular condition myosin / myorod co-filaments form [7]. The weight ratio of myosin:myorod:F-actin was 1:1:2 in both types of actomyosins.

### Phosphorylation of myorod

Myorod was phosphorylated by smMLCK as described previously [16]. Briefly, 3 μM of smMLCK, 7 μM of calmodulin (Sigma), 0.1 mM Ca^2+^ and 0.4–1 mM ATP were added to the protein in solution containing (in mM): 150 KCl, 0.5 MgCl_2_, 2 NaN_3_, 0.5 DTT, 20 imidazole-HCl, pH 7.0, and incubated for 1 hour at 25°C. Phosphorylated protein was diluted to 30 mM KCl by modified solution B with 0.5 mM MgCl_2_ and sedimented at 5000×*g* for 15 min. The pellet was re-suspended and washed three times by the same solution.

The same procedures, except the addition of smMLCK, were carried out for UnPhosMR, which was used as a control in the Mg^2+^-ATPase assay.

### Mg^2+^-ATPase assay

The Mg^2+^-ATPase activity of AM reconstituted from rabbit F-actin, rabbit or mussel myosin and various concentrations of UnPhosMR or PhosMR was determined in a medium containing (in mM): 100 KCl, 0.5 MgCl_2_, 0.5 DTT, 0.1 CaCl_2_, 10 imidazole-HCl, pH 7.2. In some cases, the co-filaments of myosin/UnPhosMR or myosin/PhosMR were formed prior to the measurement of ATPase activity by mixing the proteins in high ionic strength solutions followed by a decrease of the ionic strength as described above. The ATPase reaction was started after 10 min of incubation at 25°C by adding Mg^2+^-ATP to 0.4 mM and was terminated after 10 min by adding trichloroacetic acid to 5 mM. The amount of P_i_ liberated was evaluated colorimetrically at 625 nm by the slightly modified method of Fiske and Subbarow as described [12]. Results of ATPase assay are reported as means ± SEM with 3 to 5 different measurements.

### Superprecipitation and clearing of actomyosin

Superprecipitation and clearing of actomyosin (AM) were recorded for changing optical density of actomyosin suspension by a laser diffraction particle sizer 3600E (Malvern) in small-angle spectrophotometer mode at 633 nm. The reaction was run by adding 0.5 mM ATP to AM suspension containing: F-actin, 0.2 mg/ml; myosin, 0.1 mg/ml; myorod, 0.1 mg/ml, in solution containing (in mM): 100 KCl, 0.2 EGTA, 0.5 MgCl_2_, 1 NaN_3_, 20 Tris-HCl, pH 7.2. These conditions simulate the relaxation medium which reduced affinity of myosin to actin [20]. Dissociation of actomyosin bridges in this condition leads to a clearing, meaning a decrease in optical density of actomyosin suspension.

## Results

### Effect of myosin and twitchin contaminates in crude myorod preparation on mechanochemical cycle of actomyosin

Crude myorod fraction obtained at 33–43% ammonium saturation contained admixtures of myosin and twitchin (**Fig 1**). To obtain a pure protein this fraction was applied to a gel filtration chromatography, where three main peaks were collected (**Fig 1A**), pooled accordingly to SDS-PAGE (**Fig 1B**) and extensively dialyzed. Combined fraction 1 (CF1, enriched in myosin and twitchin), combined fraction 2 (CF2, enriched in myorod with some amount of twitchin and myosin) and combined fraction 3 (CF3, contained pure myorod) were then used in standard Mg^2+^-ATPase assay.

**Fig 2:**
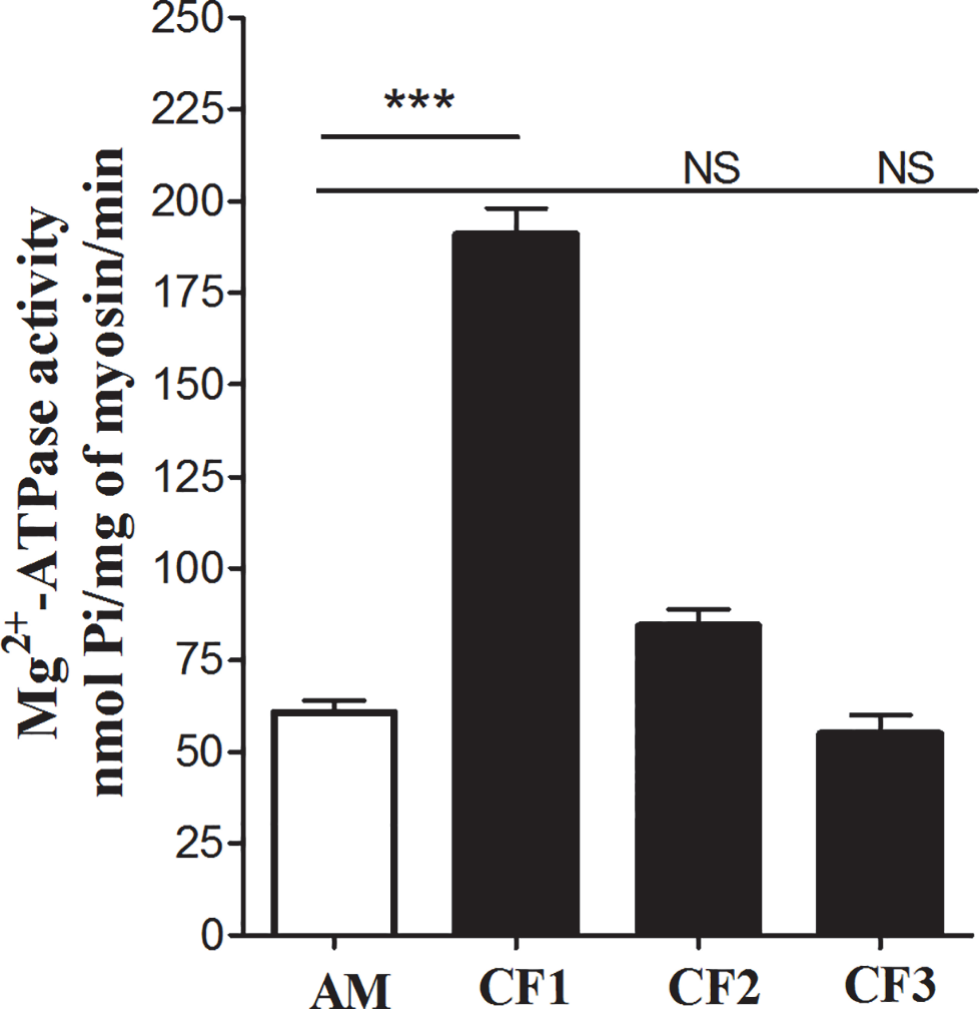
Effect of the combined fractions (CF1, CF2 and CF3) collected from the Sepharose CL 6B gel-filtration chromatography on the actin-activated Mg^2+^-ATPase activity of myosin. Note, that the CF3 fraction containing pure unphosphorylated myorod did not affect Mg^**2+**^-ATPase activity of myosin. Concentration used: mussel myosin, 0.1 mg/ml; rabbit F-actin, 0.2 mg/ml; CF1, CF2, CF3, 0.1 mg/ml. Each column represents the mean of results from three experiments ± SEM, one-way ANOVA, *:p<0.05.

CF1 strongly increased Mg^2+^-ATPase activity of AM formed from skeletal myosin and F-actin, whereas CF2 activated myosin’s ATPase significantly less than CF1 (**Fig 2**). Finally, CF3 containing pure myorod did not activate myosin’s ATPase activity at all (**Fig 2**). Thus, we confirmed that pure unphosphorylated myorod did not potentiate Mg^2+^-ATPase activity of skeletal or molluscan [7] myosins.

### Phosphorylated myorod enhances the Mg^2+^-ATPase activity of myosin if co-polymerized with myosin filaments

We recently found that presumably myorod N-terminal phosphorylation increases the actin-activated Mg^2+^-ATPase activity of myosin in the natural complex of molluscan thick filament proteins consisting of twitchin, myosin and myorod [17]. These data suggest that there are conditions under which myorod modulates the actin–myosin interaction in a phosphorylation-dependent manner.

**Fig 3:**
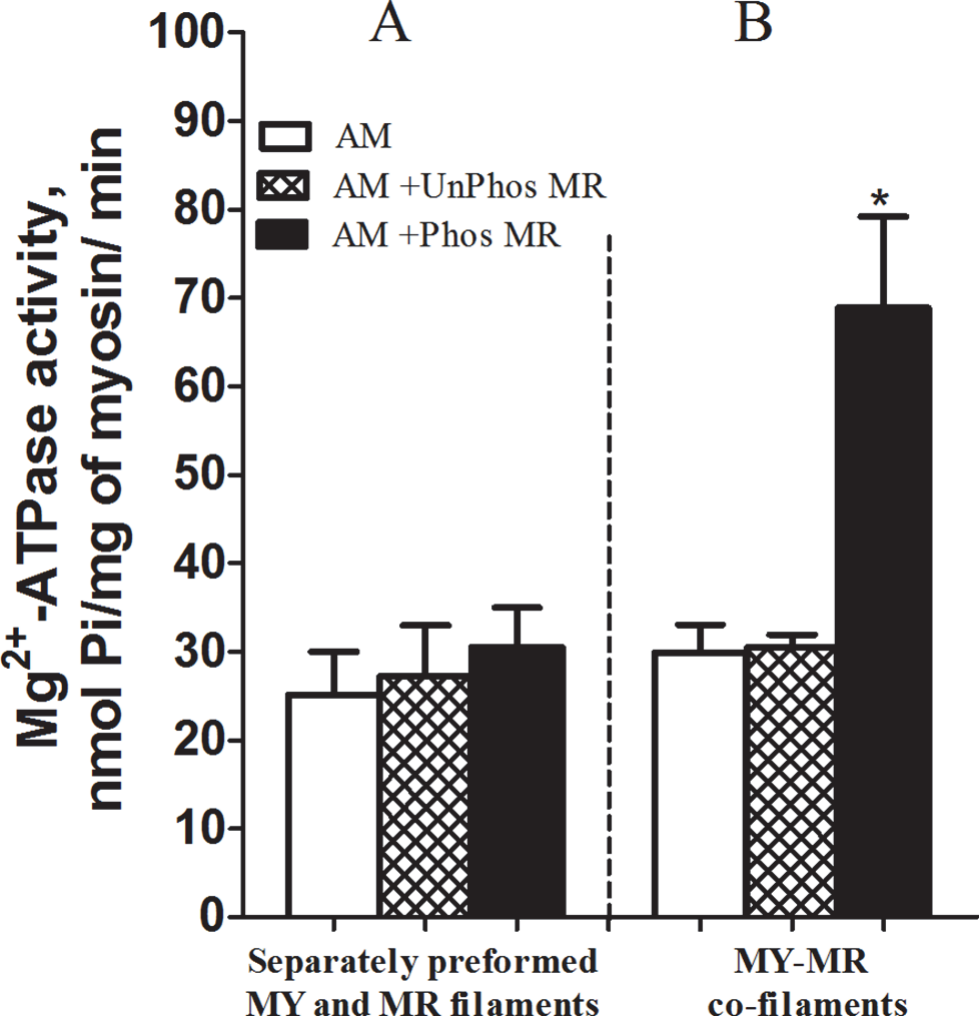
Influence of phosphorylation of myorod on the actin-activated Mg^2+^-ATPase activity of actomyosin reconstituted from separately preformed myosin and myorod filaments (A) and myosin and myorod co-filaments (B). Concentration used: mussel myosin, 0.1 mg/ml; rabbit F-actin, 0.2 mg/ml; UnPhosMR and PhosMR, 0.1 mg/ml. Each column represents the mean of results from three experiments ± SEM, one-way ANOVA, *:p<0.05.

To better understand the mechanism of such a modulation, we tried to reproduce this effect using the purified proteins: UnPhosMR and PhosMR (CF3), rabbit or molluscan myosins and rabbit F-actin. As it is shown in Fig 3A, addition of separately preformed filaments of unPhosMR or PhosMR to the reconstituted AM with preformed myosin filaments had no effect on the myosin’s Mg^2+^-ATPase activity (**Fig 3A**).

Next, the mode of actomyosin formation was changed. Myosin, UnPhosMR or PhosMR and F-actin were mixed in a high ionic strength solution (500 mM KCl) where myosin and myorod were in monomeric form and subsequently assembled into the co-filaments by quickly decreasing the ionic strength. The resulting complex was examined using the Mg^2+^-ATPase assay. Again, UnPhosMR did not activate Mg^2+^-ATPase activity of myosin, whereas PhosMR significantly increased the Mg^2+^-ATPase activity of myosin (**Fig 3B**).

**Fig 4:**
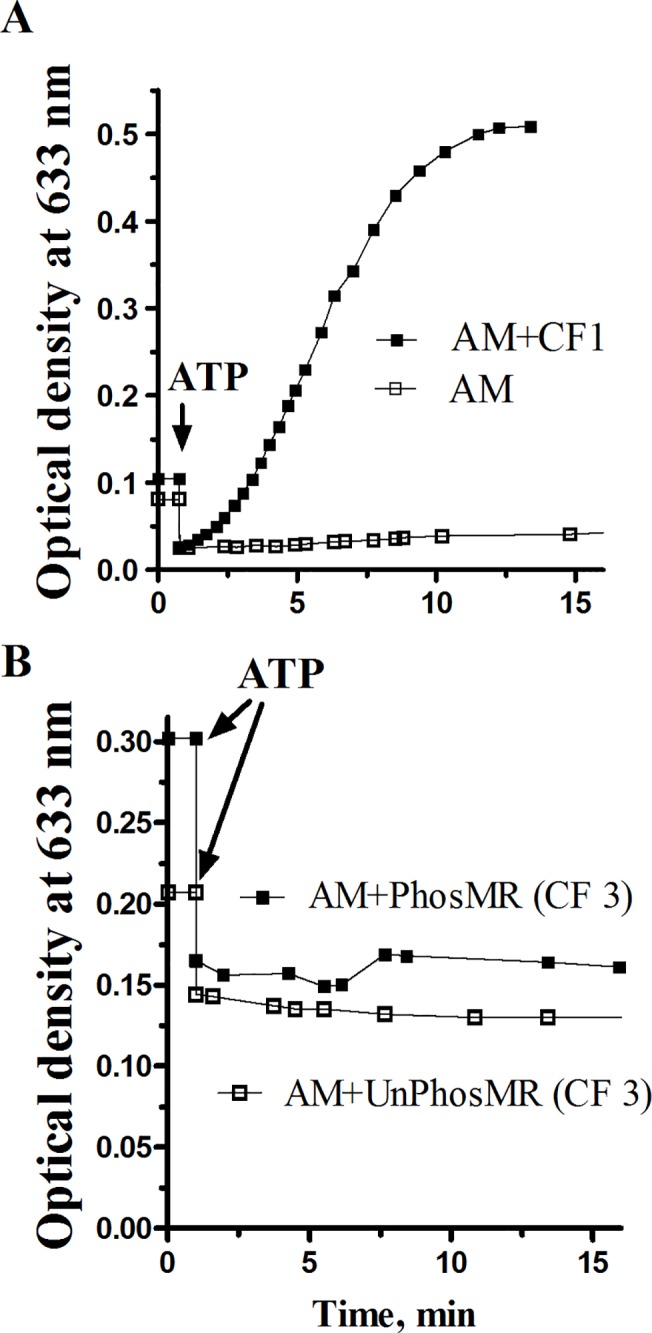
Superprecipitation and clearing of reconstituted actomyosins in the presence of CF1 (enriched with inactivated myosin and twitchin) and CF3 (pure myorod). A. AM, actomyosin (control); AM+CF1 (effect of myosin and twitchin contaminates). B. AM-PhosMR, co-filaments of myosin / phosphorylated myorod; AM-UnPhosMR, co-filaments of myosin / non-phosphorylated myorod. Concentration used: F-actin, 0.2 mg/ml; myosin, 0.1 mg/ml; myorod, 0.1 mg/ml.

Although purified PhosMR modulates myosin’s ATPase activity, it did not affect the mechanical properties of the AM either by itself or in the state of co-assembled into filaments with myosin at “clearing” condition, i.e. under conditions of reduced affinity of myosin to actin (**Fig 4B, AM-PhosMR and AM-UnPhosMR**). On the other hand, the addition of CF1 fraction (inactivated by urea myosin and twitchin) stimulates superprecipitation of actomyosin (**Fig 4A**, curve AM + CF1) because these proteins form non-dissociated cross-links with actin.

### Mg^2+^-ATPase activity of myosin at the formation of myosin / myorod co-filaments

The hypothesis that co-polymerization of myosin and PhosMR is required for the alteration of myosin’s ATPase activity was verified in a series of experiments in which myosin / myorod co-filaments were formed at different molar ratios of myorod to myosin for a constant myosin concentration.

**Fig 5:**
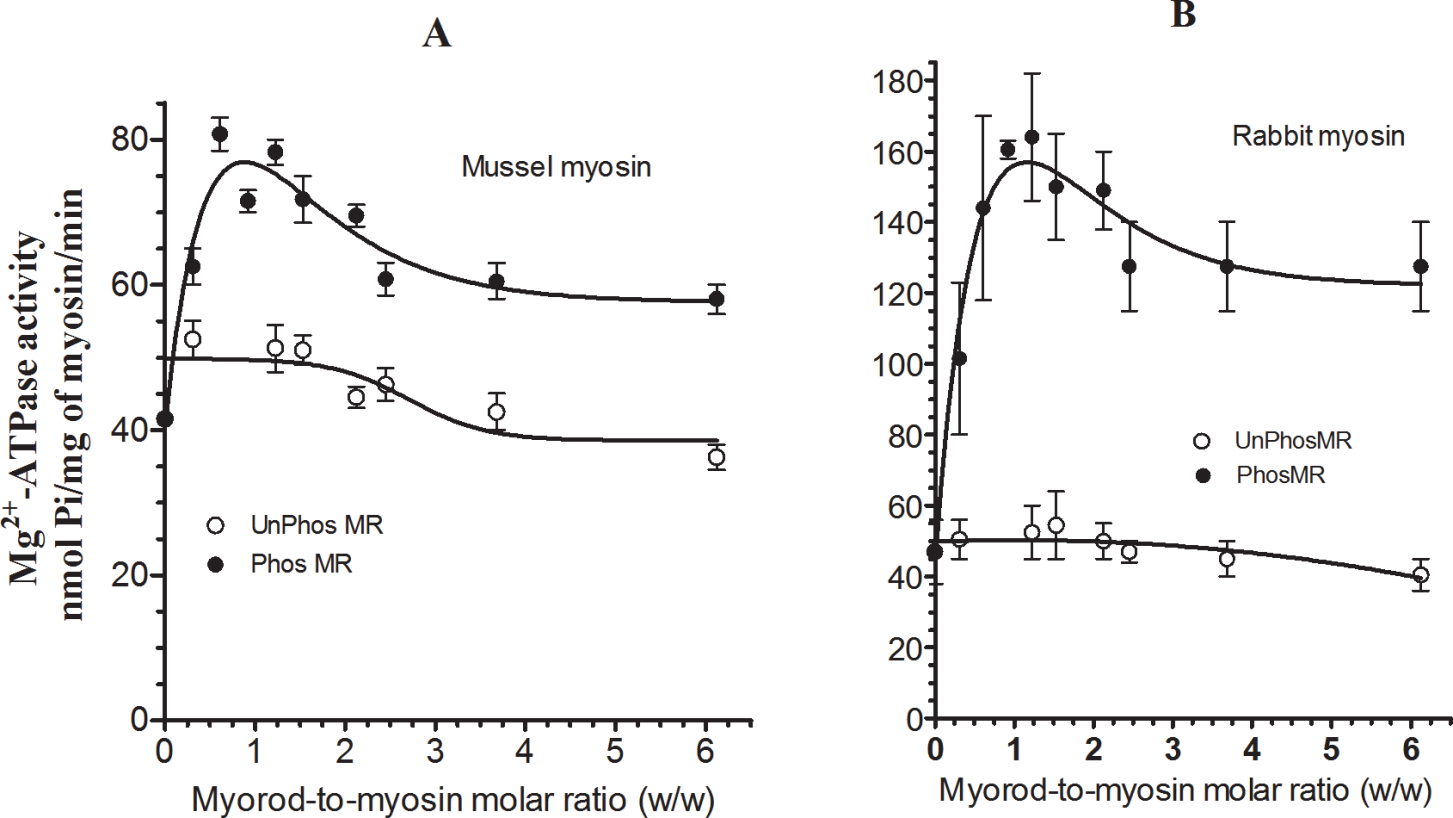
Actin-activated Mg^2+^-ATPase activity of molluscan (A) and rabbit (B) myosin co-assembled in the presence of UnPhosMR or PhosMR at different molar ratios. The ATPase activity was measured in 100 mM KCl, 0.5 mM MgCl_2_, 0.5 mM DTT, 0.1 mM Ca^**2+**^, 10 mM imidazole, pH 7.2. Concentration used: mussel and rabbit myosins, 0.1 mg/ml; rabbit F-actin, 0.2 mg/ml; UnPhosMR and PhosMR, (0.025–0.5 mg/ml). Curves of five experiments are shown.

As shown in **Fig 5A** phosphorylated myorod caused two-fold increase of actin-activated Mg^2+^-ATPase of myosin for a 1:1 PhosMR / myosin ratio. A higher ratio of PhosMR to myosin leads to a reduction in activity. In contrast, the ATPase of myosin assembled into co-filaments with UnPhosMR remained unchanged up to a 2:1 myorod: myosin ratio; the activity was inhibited by a further increase of the ratio. PhosMR also affected the ATPase activity of rabbit myosin in a similar way as of molluscan myosin (**Fig 5B**).

## Discussion

### Myorod-myosin co-filament formation is important for modulation of myosin’s motor function

In molluscan catch muscle myorod is located on the surface of the paramyosin core of thick filaments [1, 2, 4], most likely forming a complex with myosin and twitchin *in vivo*. Myosin and myorod have identical C-terminal parts [8], with the same ACD domain responsible for the filament assembly [10], and can interact *in vitro* to form co-filaments [7]. It was also shown that trace admixtures of myosin in myorod preparations as well as addition of twitchin to myorod drastically alters the structure of resulting filaments [7, 11].

In the current study we confirmed our previous conclusion that pure UnPhosMR does not activate myosin’s actin-activated Mg^2+^-ATPase (**Figs 2, 3 and 5**). In addition, we showed that PhosMR, if co- assembled into filaments with myosin, is able to potentiate the Mg^2+^-ATPase activity of myosin (**Fig 5**). This suggests that the properties of the co-filaments were determined not only by the identical C-terminal domains of myosin and myorod, but also by the N-terminus of myorod and, moreover, by its state of phosphorylation. It seems that the potentiation of the myosin activity requires an additional interaction of the phosphorylated N-terminal domain of myorod with myosin heads or the S-2 fragment. This finding is consistent with the higher affinity of a synthetic phosphorylated N-terminal peptide of myorod for myosin filaments compared to that of the unphosphorylated peptide [13].

With increasing concentration of PhosMR, the Mg^2+^-ATPase activity of myosin enhanced rapidly and achieved the maximum at equal molar ratio of PhosMR-to-myosin (**Fig 5**). We suppose that this elevated activity is determined by the maximum number of additional contacts between myorod and myosin that is established at an equimolar ratio between the proteins assembled into the co-filaments. It could alternatively be achieved through conformational changes within the myosin head or its S-2 fragment, since data obtained from experiments on mechanical properties of AM do not support the increasing of actin-myosin interaction in the presence of PhosMR (**Fig 4**) as it was shown for twitchin [12].

The inhibition of actin-activated Mg^2+^-ATPase of myosin resulting from a further increase of myorod content (2:1 myorod:myosin and higher) might also occur due to adjacent myosin molecules being more ordered with higher ratio of myorod in co-filaments. In this scenario, the binding and cycling of one myosin head on actin might exert a force on adjacent myosin-actin cross-bridges, which could inhibit their ATPase activity. A similar inhibitory effect on the Mg^2+^-ATPase activity of myosin with increasing myosin rod content in co-filaments was observed by Stepkowski et al. [21].

It should be noted that the potentiation of myosin’s ATPase in the presence of PhosMR does not depend on the source of myosin during the formation of AM. It is likely that molluscan myosin does not specialize for an interaction with myorod. However, the activating effect was more pronounced in co-filaments assembled from rabbit myosin and PhosMR (**Fig 5**).

### Does myorod participate in the catch-phenomenon?

The current understanding of the catch mechanism is based on the phosphorylation-dependent interaction of twitchin with actin without the participation of additional proteins [3, 12, 18, 15, 22, 23]. However, twitchin is a minor protein of the catch muscle [15] and its content may not be sufficient for the development of catch state. Moreover, twitchin is widely spread in different muscles that are not related to the catch contraction [5], while myorod is expressed exclusively in molluscan catch muscle [4, 5].

Also, N-terminal synthetic peptides as well as full-length myorod interact with F-actin and thin-filaments in phosphorylation- and Ca^2+^-dependent modes, respectively [13]. Myorod is phosphorylated by smMLCK at its N-terminus at the Thr141 position [16] and to a lesser extent by twitchin [17] which contains a MLCK-domain [14]. It was also shown that myorod as well as myosin are phosphorylated by Ca^2 +^-dependent myosin-associated kinase at their C-terminal parts [17, 24] in the non-helical C-terminal tailpiece [25], most likely at the Ser1693 residue [8] which corresponds to Ser973 in myorod [24]. Myosin-associated kinase is active only in the absence of calcium ions [17, 24, 25] and therefore might be important for catch development [25].

The phosphorylation of myorod at the two different sites of the molecule indicates the possibility of regulation of its unknown function. For example, twitchin is seems to be a force-sensitive kinase, which is thought to be activated by stretch that results in its autophosphorylation [26]. The idea that twitchin is a mechanical link interconnecting thin and thick filaments [12] though its interaction with actin [18] and myosin [12; 27] or myorod and paramyosin [12] supports this possibility. Stretching of muscle might trigger a chain of events responsible for N-terminal phosphorylation of myorod and potentiating Mg^2+^-ATPase of myosin during the contraction. The mechanism how phosphorylation activates myorod might include changing of the conformation of the N-domain or rupture of links between N-terminal domains of myorod similar to the dissociation of the two heads of myosin during phosphorylation [28].

In this study, we found a functional link between phosphorylation of the N-terminus of myorod and the level of actin-activated ATPase activity of myosin. This is an example of signal transmission along myorod-myosin complex affecting mechano-chemical features of myosin. However, the function of myorod is unlikely explicable solely based on properties of the myorod—myosin complex. Rather, the myorod—myosin complex is an integral part of the thick filament structures consisting of paramyosin, myosin, myorod and twitchin. Nevertheless, the results of this study clearly indicate that myorod is not simply a structural component of thick filaments but may act as an active player of catch muscle contraction.
